# Supplementary material for: Breeding French bulldogs so that they breathe well—A long way to go
Source: PLoS One. 2019 Dec 16;14(12):e0226280. doi: 10.1371/journal.pone.0226280 (PMC6913956; doi:10.1371/journal.pone.0226280)
Supplement: Attachment — Submitted filename: Response to Reviewers.docx [file pone.0226280.s005.docx]

6. Review Comments to the Author

Reviewer #1: the insistence that brachycephalic obstructive airway syndrome is best evaluated by direct visual examination misses the facts that a. this is a functional disease b. there is no current scheme to score CT/ endoscopy lesions with disease severity. Please adjust lines 59 and 319 -321 accordingly.

I think the authors are stating that there is a relationship between bfs and bs but they do not clearly define bs. From the study it seems that bs is a composite of various external conformational measurements. If this is the case can this be clearly stated.

*Thank you for this comment. We agree that some key definitions and explanations were missing:*

*In line 16-19 we have explained more precisely what BS is (ie. Brachycephalic syndrome (BS) is a pathophysiological disorder caused by excessive soft tissue within the upper airways of short-nosed dog breeds, causing obstruction of the nasal, pharyngeal and laryngeal lumen and thereby giving rise to various symptoms such as respiratory distress). In line 61 to 71 we have clarified why a functional assessment is a reasonable proxy measure for BS.*

Can the authors address the weak r results

*We have addressed the r results further in line 310-314*

Line 277. this study does not 'establish' - there is no validation of the bfs. preferable to use 'introduce' to avoid over confidence in an as yet unproven system. This is particularly the case as a new (and interesting) decibel recording system has been used which needs further investigation before it is advocated as a grading scheme

*We have rephrased this sentence (line 307-308): In the present study we have* ***introduced the calculation of BSF scores****, on a continuous scale, that rely on recordings of upper airway noise and decibel recordings before, during, and after exercise to assess the degree of BS*

Reviewer #2: The title, although improved, remains in need of editing. The intent of the title is to convey that through concerted selection practices targeting BS can reduce the prevalence of BS. The current title of “Breeding French bulldogs with unobstructed breathing – possible but a long way to go” while catchy is still not informative. Of course it is possible to breed French bulldogs with unobstructed breathing. That however is not the intent of the manuscript. The manuscript is demonstrating that although French bulldogs with unobstructed breathing do exist, they are not that prevalent in the population the authors used and to increase the proportion of French bulldogs in a population that do not have BS it will take concerted selection and time in order to maintain genetic diversity. A more accurate title would be something along the lines of that conveys the concept that concerted selection can reduce obstructed breathing in the French bulldog but will take time.

*We have changed the title so that it now reads: Breeding French bulldogs so that they breathe well - a long way to go.*

Line 78 “Exercise testing is used both in humans [7, 8] and dogs [9] to measure reduced functional ability.” Please define what is meant by “functional ability”.

*Exercise testing has been explained in more detail and with more appropriate references (see Line 104-112)*

Line 164 should be microsatellites.

*Spelling has been corrected (line 200)*

Line 268-269. The authors use reference 9 to illustrate the effect of BS on health and welfare. However the study detailed in reference 9 did not evaluate brachycephalic dogs. Thus this statement still is in need of references to demonstrate that “several studies….”

*We apologize for not providing sufficient documentation for this statement. Three more appropriate references have been provided (line 305 and 307)*

Line 268-269: Additionally the sentence needs editing as it appears something is missing: “..several studies have been performed to evaluate the severity of BS.” It would appear that the authors are tying together severity of BS and an impact on health or function.

*We have included ‘****based on exercise tests’****(line 307)to make the statement more comprehensible.*

Line 272: should be reflect the severity of BS (not reflect upon).

*We have removed ‘upon’ as suggested*

Line 272-273: comma needed after characterization “To lend support to the functional characterization, efforts have also been made to identify the…”

*Thank you: we have included ‘,’ as suggested (line 316)*
